# Gold Nanorods Embedded in Mesoporous Silica for Photothermal Therapy and SERS Monitoring in T47D Breast Cancer Cells

**DOI:** 10.3390/pharmaceutics18030310

**Published:** 2026-02-28

**Authors:** Annel Armenta-Gamez, Alejandro Pedroza-Montero, Alejandra Tapia-Villasenor, Erika Silva-Campa, Hector Loro, Rodrigo Melendrez, Sergio A. Aguila, Karla Santacruz-Gomez

**Affiliations:** 1 PhD Program in Nanoscience, Center for Scientific Research and Higher Education of Ensenada (CICESE), Carretera Ensenada-Tijuana No. 3918, Zona Playitas, Ensenada 22860, Baja California, Mexico; annelc@ens.cnyn.unam.mx; 2 Physics Department, University of Sonora, Blvd Luis Encinas y Rosales S/N, Col. Centro, Hermosillo 83000, Sonora, Mexico; francisco.pedroza@unison.mx; 3 PhD Program in Nanotechnology, Physicas Deparment, University of Sonora, Blvd Luis Encinas y Rosales S/N, Col. Centro, Hermosillo 83000, Sonora, Mexico; aletapia92@gmail.com; 4 Department of Physics Research, University of Sonora, Blvd Luis Encinas y Rosales S/N, Col. Centro, Hermosillo 83000, Sonora, Mexico; erika.silva@unison.mx (E.S.-C.); rodrigo.melendrez@unison.mx (R.M.); 5 Department of Physics, Faculty of Sciences, National University of Engineering, Tupac Amaru 210, Rimac, Lima 100190, Peru; hloro@uni.edu.pe; 6 Center for Nanosciences and Nanotechnology, National Autonomous University of Mexico, Ensenada 22860, Baja California, Mexico; aguila@ens.cnyn.unam.mx

**Keywords:** gold nanorods, mesoporous silica nanoshells, plasmonic photothermal therapy, surface-enhanced raman scattering, breast cancer, nanotheranostics

## Abstract

**Background:** The development of plasmonic photothermal therapy (PPTT) to trigger cancer cells is often hindered by uncontrolled overheating and the lack of real-time feedback. **Methods:** In this study, we report the synthesis of gold nanorod-embedded mesoporous silica nanoshells (AuNR@Si) as a multifunctional theranostic platform designed for controlled hyperthermia and surface-enhanced Raman spectroscopy (SERS) monitoring. Using a layer-by-layer templating strategy, AuNRs were successfully obtained within a hollow silica architecture. **Results:** While AuNRs alone exhibited rapid photothermal spikes reaching 64 °C, the AuNR@Si platform moderated the photothermal response, maintaining a stable therapeutic window (41–45 °C). In vitro assays using T47D breast cancer cells demonstrated a 33% reduction in viability following irradiation. Furthermore, the structural stability of the AuNR@Si platform enabled SERS monitoring of cellular damage, identifying specific biochemical fingerprints of protein denaturation, cytochrome c release and DNA fragmentation. **Conclusions:** These results suggest that AuNR@Si nanoshells provide a safer, regulated approach to photothermal ablation with the added benefit of molecular detection, demonstrating proof-of-concept theranostic functionality in a luminal breast cancer model.

## 1. Introduction

Gold nanorods (AuNRs) exhibit unique plasmonic properties that facilitate a range of oncological applications, including image-guided interventions, real-time molecular diagnostics, and light-triggered therapeutic interventions. Due to their anisotropic geometry, AuNRs support two distinct localized surface plasmon resonance modes [1]: a transverse mode (T-LSP) in the visible spectrum and a longitudinal mode (L-LSP) in the near-infrared (NIR) region. The L-LSPR can be precisely tuned within the so called “biological transparency window” (700–900 nm) [2], a spectral region where endogenous tissue chromophores, such as hemoglobin, melanin, and water, exhibit minimal absorption and scattering [3]. Operation within the first biological window enables enhanced tissue penetration and efficient energy delivery while substantially mitigating the photochemical damage typically associated with higher-energy visible irradiation [4]. This combination of deep penetration, controlled energy conversion, and biocompatible activation makes gold AuNRs particularly attractive for biomedical applications [5], including bioimaging, plasmonic photothermal therapy and SERS, where non-invasive and biologically safe excitation is essential.

Despite their outstanding potential as nanoagents, AuNRs present intrinsic limitations that restrict their direct use in biological environments. Their synthesis typically relies on cytotoxic surfactants such as cetyltrimethylammonium bromide (CTAB) [6] which disrupt cellular membranes and must be rigorously removed or effectively shielded prior to in vitro or in vivo application. In addition, AuNRs are susceptible to morphological reshaping or melting under laser irradiation [7], a process that alters their anisotropic geometry, dampens their LSPR, and consequently compromises their photothermal performance and spectral stability. These physicochemical vulnerabilities not only affect therapeutic efficiency but also undermine reproducibility and long-term functionality.

To overcome these challenges, the integration of AuNR into stable and biocompatible carriers, such as silica [8], chitosan [9], polyethylene glycol (PEG) [10], liposomes [11] and stimuli-responsive polymer matrices, has emerged as a central design strategy. Such hybrid architectures enhance colloidal stability, mitigate surfactant-related cytotoxicity, improve biological compatibility and protect the plasmonic core against thermally induced deformation [12]. As reported by Arcos Rosero et al. [13], and further discussed in recent reviews on hybrid plasmonic architectures [14], such engineered coatings and composite designs not only enhance physicochemical stability, but also preserve the intrinsic plasmonic activity of gold nanoshells under therapeutic irradiation.

Among these platforms, silica-based matrices stand out as one of the most widely adopted and versatile supports, owing to their chemical inertness, tunable porosity, optical transparency, and proven biocompatibility [8]. However, embedding anisotropic nanoparticles such as AuNRs into silica matrices remains considerably more challenging than incorporating spherical gold nanoshells. Unlike isotropic nanospheres, AuNRs exhibit facet-dependent surface chemistry and heterogeneous charge distributions along their longitudinal and transverse axes [15]. These anisotropic features give rise to orientation-dependent electrostatic and van der Waals interactions, which hinder uniform adsorption on templates and promote preferential end-binding or side-binding during assembly. As a result, AuNRs display an increased propensity for aggregation and rotational disorder during sol–gel growth, phenomena that are rarely encountered with spherical particles [16]. Moreover, the anisotropic geometry of AuNRs renders them highly sensitive to interfacial stresses and local dielectric changes, such that conventional silica-coating protocols—originally developed for isotropic colloids [8]—often lead to nonuniform coverage, partial embedding, plasmon damping, or even morphological reshaping [17]. These limitations underscore the need for controlled templating strategies capable of stabilizing rod orientation, regulating surface charge, and decoupling silica growth from rod morphology in order to preserve the NIR plasmonic functionality of AuNRs. The present study addresses these challenges through a reproducible, template-assisted approach that enables the integration of AuNRs into mesoporous silica nanoshells while preserving their plasmonic properties. This architecture maintains dual plasmonic functionality, allowing simultaneous photothermal activation and SERS-based molecular sensing. Using T47D breast cancer cells as a model system, we demonstrate that these AuNR@Si nanoshells mediate NIR-induced photothermal damage while simultaneously enabling SERS-based detection of the associated biochemical alterations at the molecular level.

## 2. Materials and Methods

Carboxylate-modified polystyrene (PS-COOH) spheres (50 and 200 nm diameter; Cat #15913 and #08216, respectively) were purchased from PolySciences (Warrington, PA, USA). Poly(diallyl dimethylammonium chloride) (PDDA, 28% in water; Cat #409014), chloroauric acid trihydrate (HAuCl_4_·3H_2_O, 99.9%; Cat #520918), tetrakis(hydroxymethyl)-phosphonium chloride (THPC, 80% solution in water; Cat #404861) and tetraethyl orthosilicate (TEOS; Cat #131903) ammonium hydroxide (NH_4_OH, 29%; ACS reagent; Cat #320145), dimethylformamide (DMF; 99.8% Cat #227056), 4-mercaptobenzoic acid (4-MBA, Cat #662543), anhydrous ethyl alcohol (EtOH; Cat #459844), penicillin-streptomycin (Cat #P4333), and thiazolyl blue tetrazolium bromide (MTT, ≥97.5%, Cat #M5655) were purchased from Merck (Darmstadt, Germany). Sodium hydroxide (NaOH; Cat #3722) was purchased from J.T. Baker (Phillipsburg, NJ, USA). Isopropyl alcohol (IPA; Cat #06091) was purchased from Fermont (Monterrey, Mexico). Ascorbic acid (AA, Cat #0060) was purchased from Meyer (Mexico City, Mexico). Dulbecco’s modified eagle medium (DMEM, Cat #31966047) and fetal bovine serum (FBS, Cat #10437028) were purchased from Thermo Fisher Scientific (Waltham, MA, USA). Deionized (DI) water was obtained by a Millipore Direct-Q 3 UV (18.2 MΩ resistance) from Merck (Darmstadt, Germany).

### 2.1. Synthesis of AuNR@Si Nanoshells

Step 1. Preparation of the core–satellite PS template (PS-Pollen). Core–satellite PS templates were generated using 200 nm carboxylated PS spheres. A volume of 0.5 mL of a 2.5 wt% PS suspension was mixed with 3.0 mL DI water and 2.5 mL of 1 wt% PDDA and stirred for 20 min to invert the native surface charge. The coated PS spheres were purified by centrifugation (10,000 rcf, 45 min) three times with DI water and twice with EtOH and subsequently redispersed in 5 mL of 80% EtOH. To assemble the satellite layer, 150 μL of 50 nm PS spheres (pre-diluted in 850 μL DI water) were added to the core suspension and incubated overnight at 4 °C. The resulting “PS-pollen” templates were then UV-irradiated (254 nm) to promote surface activation for gold nanoparticle attachment.

Step 2. AuNR synthesis and embedding. AuNRs were synthesized using a silver-assisted, CTAB-mediated seeded-growth method. The seed solution (3.5 mL) was obtained by adding 8 μL of 0.1 M HAuCl_4_ and 20 μL of freshly prepared, ice-cold 0.1 M NaBH_4_ to a mixture containing 1.66 mL of 0.2 M CTAB and 1.84 mL ultrapure water. The growth solution (10.8 mL) was formulated by combining 5.0 mL of 0.2 M CTAB with 5.5 mL ultrapure water, followed by sequential addition of 50 μL of 0.1 M HAuCl_4_, 60 μL of 0.01 M AgNO_3_, and 55 μL of 0.1 M AA. Nanorod growth was initiated by introducing 12 μL of the seed solution under gentle mixing. CTAB-stabilized AuNRs (positively charged) were coated with poly(sodium p-styrenesulfonate) (PSS) by mixing equal volumes of the AuNR suspension and a 0.01 M PSS solution and incubating the mixture for 1 h. The resulting AuNR–PSS nanoshells were recovered by centrifugation, redispersed in ultrapure water, and displayed a net negative surface charge, as confirmed by zeta potential measurements. UV-activated PS templates were dispersed in 5 mL of solution and mixed with 2.5 mL of AuNR suspensions. The mixture was incubated overnight at 60 °C to promote electrostatic binding of the nanoparticles onto the “PS-pollen” surface. Excess AuNRs were removed by two centrifugation cycles (3200 rcf).

Step 3. Silica deposition and template removal; layer-by-layer silica coating. A dual silica nanoshell was grown via stepwise hydrolysis and condensation of TEOS. The AuNR@PS pellet was resuspended in a mixture of IPA, water, and NH_4_OH, after which 4.5 mL of diluted TEOS was added dropwise and allowed to react for 20–24 h (first layer). After centrifugation, a second TEOS aliquot (2.25 mL) was introduced and the reaction proceeded for an additional 12 h to yield the complete silica shell. Removal of the PS template and final purification. The AuNR@PS@Si product was washed three times with EtOH and subsequently incubated in 5.7 mL DMF at 60 °C for 48 h to dissolve the PS scaffold. The resulting hollow AuNR@Si nanoshells were collected by centrifugation (8000 rcf), washed sequentially with DI water and EtOH, and stored in anhydrous EtOH.

### 2.2. Physicochemical Characterization of AuNR@Si Nanoshells

The structural and colloidal properties of the AuNR@Si nanoshells were systematically evaluated by scanning electron microscopy (SEM), dynamic light scattering (DLS), zeta potential measurements, and UV–Vis–NIR spectroscopy in order to confirm successful assembly, colloidal stability, and preservation of plasmonic functionality.

### 2.3. Photothermal Conversion Efficiency of AuNR@Si Nanoshells

The capacity of AuNR@Si to absorb NIR radiation and dissipate it as heat within the therapeutic hyperthermia range was evaluated in colloidal suspensions. AuNR@Si samples at a concentration of 50 μg/mL were placed in a quartz cuvette and irradiated using a 785 nm NIR laser at a power density of 1.2 W/cm^−2^ (spot diameter 5 mm) for 25 min. Temperature measurements were recorded using a USB TC–08 thermocouple data logger equipped with a Type K thermocouple probe from Pico Technology (Cambridgeshire, UK). After the laser was turned off, the cooling profile was monitored until the suspension returned to the ambient temperature. These heating and cooling curves were utilized to determine the photothermal efficiency as follows:

$$\eta =\frac{hS\left(T_{max}-T_{amb}\right)-Q_{dis}}{I\left(1-10^{-A_{785}}\right)}$$

where h is heat transfer coefficient, S is surface area of the container, T_max_ is maximum steady-state temperature reached during irradiation, T_amb_ is ambient (room) temperature, Q_dis_ is heat dissipated by the solvent and container (determined from a control experiment), I is laser power incident on the sample, and A_785_ is absorbance of the AuNR@Si at 785 nm.

### 2.4. SERS Efficiency of AuNR@Si Using 4-MBA

The SERS performance of AuNR and AuNR@Si nanostructures was evaluated using 4-MBA as a probe molecule. Nanoparticle dispersions (50 µg/mL) were incubated with 4-MBA (10 mM) for 12 h to allow thiol–gold chemisorption. Excess and unbound 4-MBA were removed by centrifugation (8000 rpm, 10 min), followed by washing with DI water and redispersion of the functionalized nanoparticles. This washing step was repeated twice to ensure removal of free molecules. Subsequently, 20 µL of the 4-MBA-modified AuNR or AuNR@Si suspension was drop-cast onto CaF_2_ substrates and dried at room temperature (spot diameter ~5 mm). Raman and SERS spectra were acquired using a WITec alpha 300 RA Raman microscope (Ulm, Germany) equipped with a 532 nm continuous-wave laser, a 50× objective, 1 s integration time, and 10 accumulations under identical acquisition conditions. All spectra were baseline-corrected prior to analysis. The SERS enhancement factor (EF) was calculated using the 1595 cm^−1^ vibrational band of 4-MBA according to:

EF=ISERSNSERSIRamanNRaman

where EF is the Raman enhancement factor, I_SERS_ is the SERS intensity, N_SERS_ is the number of molecules on the laser surface, I_Raman_ is the Raman intensity, N_Raman_ is the number of excited molecules.

### 2.5. In Vitro Evaluation of PPTT and SERS Monitoring in T47D Breast Cancer Cells

The photothermal response of AuNR and AuNR@Si nanoshells was evaluated in vitro in T47D breast cancer cells cultured in DMEM supplemented with 5% FBS and antibiotics at 37 °C under 5% CO_2_. Cells were seeded at 3 × 10^5^ cells per well on CaF_2_ substrates in 12-well plates and allowed to adhere for 24 h. After incubation with AuNRs or AuNR@Si (50 µg/mL, 4 h), cells were washed, refreshed with medium, and irradiated with a 785 nm NIR laser (1.2 W/cm^2^, 5 mm spot) for 15 min. Temperature was monitored using a thermocouple. Following PPTT, cellular damage was assessed by SERS molecular fingerprint analysis (500–1800 cm^−1^) using 532 nm excitation on a WITec alpha 300 RA Raman microscope (1 s integration, 10 accumulations).

### 2.6. MTT Assays

Cell viability was evaluated using the MTT assay. T47D cells (1 × 10^4^ cells/well) were seeded in 96-well plates and incubated for 24 h prior to treatment with AuNRs or AuNR@Si (50 µg/mL), medium alone (negative control), or 10% DMSO (positive control) for 24 h. After treatment, cells were incubated with MTT solution (0.5 mg/mL) for 4 h, followed by dissolution of formazan crystals in DMSO. Absorbance was measured at 540 nm, and viability was calculated relative to untreated controls as (A_treated_/A_control_) × 100.

## 3. Results

### 3.1. Synthesis and Characterization

**Figure 1:**
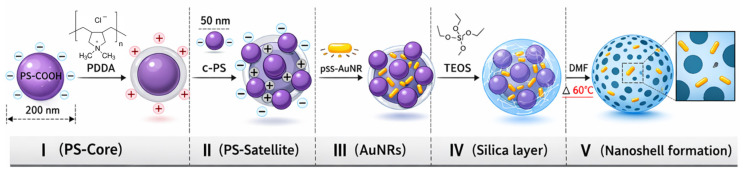
Schematic illustration of the templated synthesis of AuNR@Si nanoshells. (I) Carboxylated polystyrene (c-PS, 200 nm) spheres are cationized with PDDA; (II) smaller c-PS satellites (50 nm) are electrostatically assembled to form a pollen-like template and UV-conditioned to generate surface vacancies; (III) AuNRs are immobilized at the active sites; (IV) a silica shell is formed via TEOS hydrolysis–condensation; and (V) polymer removal in DMF at 60 °C yields hollow mesoporous silica nanoshells with embedded AuNRs.

Gold nanorod-embedded mesoporous silica nanoshells (AuNR@Si) were successfully fabricated using an adapted layer-by-layer templating strategy [18]. The step-by-step synthesis of AuNR@Si nanoshells is schematically illustrated in Figure 1. Anionic AuNRs were electrostatically immobilized onto a cationized, UV-conditioned polystyrene template, followed by silica growth via TEOS hydrolysis–condensation and removal of the polymeric core to yield hollow nanoshells. Zeta-potential analysis confirmed sequential surface-charge inversion and colloidal stability at each assembly stage, evidencing controlled AuNR incorporation and reproducible nanoshell formation [19]. The resulting AuNR@Si nanoshells exhibit well-defined hollow architectures with AuNRs embedded at the silica surface, establishing a structurally confined plasmonic platform suitable for dual optical functionality.

**Figure 2:**
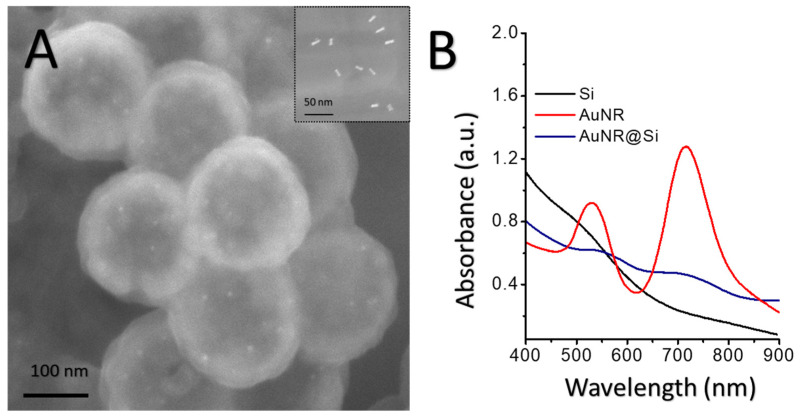
Structural and optical characterization of gold–silica hybrid nanoshells. (**A**) SEM image of AuNR@Si nanoshells displaying AuNRs (inset) incorporated into the silica matrix, and (**B**) their UV–Vis–NIR absorbance spectra.

Zeta-potential analysis confirmed controlled surface-charge inversion throughout the layer-by-layer assembly, validating each fabrication step and ensuring reproducible AuNR incorporation. The carboxylated polystyrene template exhibited a strongly negative potential (−60.7 ± 2.4 mV), which reversed upon PDDA adsorption (+36.2 ± 2.0 mV), while PSS-modified AuNRs displayed an anionic surface (−39.0 ± 1.0 mV), enabling their electrostatic immobilization. Following silica growth and template removal, the resulting AuNR@Si nanoshells exhibited moderately negative zeta potentials (−22.1 ± 4.1 mV), consistent with deprotonated silanol groups and stable aqueous dispersion. Electron microscopy (Figure 2A) revealed well-defined hollow nanoshells with uniform silica thickness, minimal aggregation, and AuNRs (31 ± 3 nm × 10 ± 2 nm) localized at the nanoshell surface, yielding an average particle diameter of 193 ± 12 nm. Correspondingly, optical extinction spectra (Figure 2B) displayed the characteristic dual plasmonic response of AuNR, with a transverse localized surface plasmon (T-LSP) at 536 ± 3 nm and a pronounced longitudinal mode (L-LSP) at 710 ± 1 nm. The preservation of the visible T-LSP and the NIR-shifted L-LSP underscores the structural integrity of the embedded AuNRs and supports their suitability for combined SERS sensing and NIR-mediated photothermal applications.

### 3.2. Surface Enhanced Raman Spectroscopy Efficiency in AuNR@Si Hybrid Nanoshells

**Figure 3:**
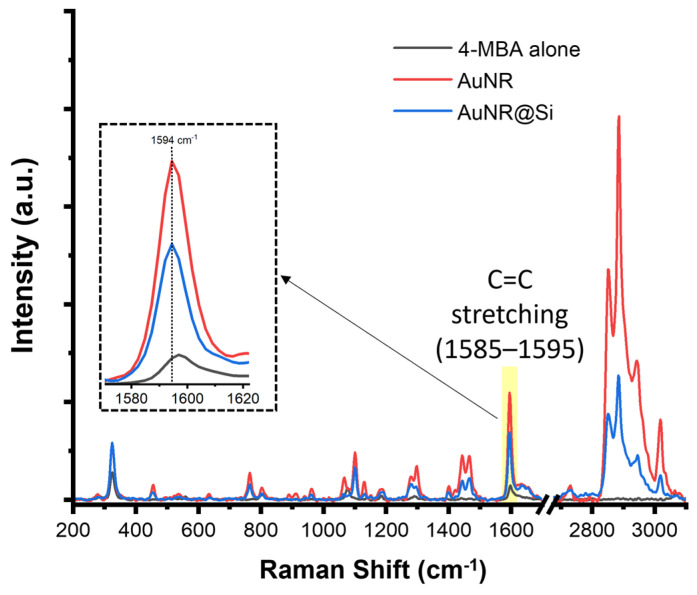
Raman and SERS spectra of 4-MBA acquired on AuNR and AuNR@Si substrates under 532 nm excitation. The spectra of 4-MBA alone (black), AuNRs (red), and AuNR@Si (blue) were recorded under identical acquisition conditions. The shaded region highlights the aromatic C=C stretching band (1585–1595 cm^−1^) used for enhancement factor (EF) calculation. The inset shows a magnified view of the 1550–1620 cm^−1^ region, where enhanced intensity is observed for both nanostructured substrates relative to the Raman reference.

To evaluate the plasmonic efficiency of the synthesized nanostructures, SERS measurements were performed using 4-MBA as a molecular probe. This characterization quantifies the electromagnetic field enhancement provided by the AuNR architectures and confirms the accessibility of the metal surface. Figure 3 illustrates the Raman spectrum of 4-MBA in comparison to the SERS signatures acquired from 4-MBA-functionalized AuNR and AuNR@Si substrates. The characteristic vibrational features of 4-MBA are preserved in all spectra, confirming stable thiol–gold chemisorption after functionalization and washing, consistent with previous reports on 4-MBA adsorption on gold surfaces [20]. A pronounced enhancement of the aromatic C=C stretching band (1585–1595 cm^−1^ region) is observed for both nanostructured substrates relative to the Raman reference. This vibrational mode is widely employed for quantitative SERS analysis due to its strong and reproducible signal in 4-MBA-functionalized plasmonic systems [21]. The inset in Figure 3 highlights this spectral region, showing the increased peak intensity for both AuNRs and AuNR@Si relative to the 4-MBA reference. The corresponding enhancement factors, calculated from the 1594 cm^−1^ band, were estimated to be 4.5 × 10^5^ for AuNRs and 9.8 × 10^3^ for AuNR@Si. The approximately two-order-of-magnitude difference in EF suggests that the AuNR@Si nanoshells may exhibit a reduced density of accessible electromagnetic hotspots or a modified local field distribution compared to the AuNRs alone [22]. Nevertheless, both platforms demonstrate sufficient analytical sensitivity to resolve the fingerprint region (400–1700 cm^−1^), validating their performance as active plasmonic substrates.

### 3.3. Plasmonic-Induced Heating of AuNR@Si Nanoshells

**Figure 4:**
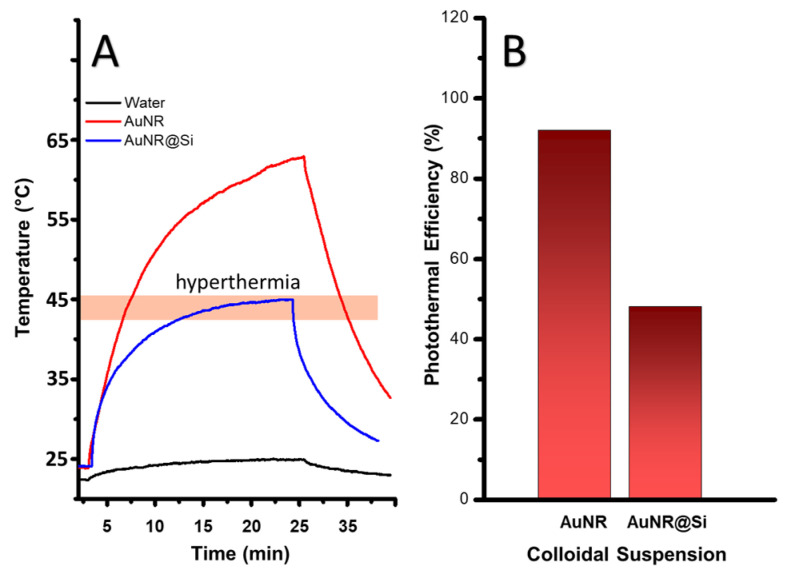
Plamonic photothermal efficiency of AuNRs and AuNR@Si. (**A**) Heating-cooling curve of water, AuNRs and AuNR@Si under 785 nm laser irradiation. (**B**) Photothermal conversion efficiency (η) of AuNRs and AuNR@Si estimated using the Roper method.

The photothermal response of the plasmonic nanosystems was assessed in vitro by exposing aqueous suspensions of AuNRs and AuNR@Si to identical irradiation conditions (λ_exc_ = 785 nm) in order to (i) quantify the intrinsic heating capability of AuNRs, and (ii) determine how this behavior is modulated by silica embedding. As shown in Figure 4A, AuNRs show a pronounced increase in temperature, reaching ~64 °C, consistent with highly efficient excitation of L-LSP mode. This rapid thermal response results in only a brief transit through the moderate hyperthermia range (41–45 °C; ~1 min), followed by continued heating well beyond therapeutically desirable levels. Such behavior reflects high photothermal power but limited thermal controllability. In contrast, AuNR@Si display a markedly attenuated and regulated heating profile. The temperature increases gradually and stabilizes in hyperthermia at ~43 °C, and remains within this window for an extended period (~15 min) under continuous irradiation.

Figure 4B and Table 1 summarize these trends. AuNRs exhibit a high apparent photothermal efficiency (~90%), consistent with efficient NIR absorption and rapid energy conversion. The lower apparent efficiency observed for AuNR@Si (~48%) may reflect a combination of factors, including thermal modulation by the silica matrix and differences in the effective plasmonic gold contribution within the composite nanoshell. Further systematic studies will be required to elucidate the relative contributions of these effects. Importantly, this reduced apparent efficiency does not diminish therapeutic relevance; rather, it corresponds to a moderated thermal response that enables controlled and sustained hyperthermia without reliance on peak temperature maximization.

### 3.4. SERS-Based Assessment of Plasmonic Photothermal Therapy (PPTT) in T47D Cells Using AuNR@Si Nanoshells

**Figure 5:**
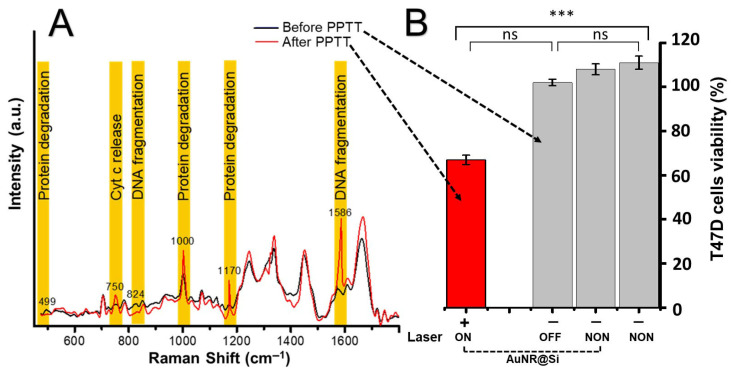
SERS monitoring of PPTT effect in a breast cancer cell line (T47D). (**A**) Representative SERS spectra of T47D cells before (black) and after (red) AuNR@Si-mediated PPTT, showing bands associated with protein degradation, cytochrome c release, and DNA fragmentation. (**B**) T47D cells viability under different conditions: control (no nanoparticles, no laser), laser only, AuNR@Si without irradiation and AuNR@Si + NIR irradiation (PPTT). No significant differences (ns) were observed for control groups, while a significant decrease in viability was detected only after combined AuNR@Si + NIR irradiation (*** *p* < 0.001).

Based on the photothermal characterization in aqueous suspension, irradiation parameters were selected to achieve moderate hyperthermia (41–45 °C) with controlled thermal dosing, particularly for the AuNR@Si system, which exhibited sustained residence within this therapeutic window and moderated photothermal efficiency. These parameters were subsequently reproduced in vitro in cell-based assays to evaluate whether the thermally optimized conditions identified in solution translate into effective cellular responses. Figure 5 presents the cellular outcomes following PPTT under these optimized conditions. Figure 5A shows comparative Raman spectra acquired from cells before and after PPTT, revealing spectral alterations after treatment. Specifically, an increase in Raman intensity is observed across bands associated with protein integrity, cytochrome c–related molecular signatures [23] and DNA structure, including regions near ~750–830 cm^−1^, ~1000 cm^−1^, ~1170 cm^−1^, and ~1580 cm^−1^. These changes are consistent with PPTT-induced molecular damage, indicating disruption of cellular proteins, mitochondrial signaling pathways, and nucleic acid integrity under the applied hyperthermia conditions (Table 2) [22,24]. The SERS-detected molecular alterations are consistent with the viability results shown in Figure 5B. T47D cells treated with AuNR@Si alone exhibited viability comparable to untreated controls, confirming biocompatibility at 50 µg/mL. Likewise, 785 nm irradiation in the absence of nanoparticles did not significantly affect cell survival, excluding non-specific photothermal effects. In contrast, a significant reduction in viability (~33%) was observed only under the combined AuNR@Si + NIR condition, supporting a nanoparticle-mediated photothermal mechanism. This level of cytotoxicity is consistent with in vitro PPTT studies performed under moderate hyperthermic conditions [25].

## 4. Discussion

Here, gold nanorods were efficiently embedded within mesoporous silica nanoshells (AuNR@Si) using a template-assisted layer-by-layer (LbL) approach combined with PPS-modified AuNRs. This with the aim to obtain a theranostic platform employing a dual-excitation (L-LSP and T-LSP modes) [26] strategy to integrate therapeutic and monitoring functions respectively. Achieving efficient AuNR incorporation into a polymeric matrix is nontrivial, as anisotropic plasmonic nanoparticles exhibit orientation-dependent interactions and a stronger propensity for aggregation compared with isotropic nanospheres [27]. In this context, the main factor contributing to successful AuNR loading appears to be the electrostatic control through charge engineering [27,28], where the LbL polyelectrolyte strategy was used to invert the initial CTAB surface charge [29], thereby regulating their surface charge and mitigating aggregation. The AuNR@Si nanoshells retain their characteristic anisotropy and dual plasmonic behavior, as evidenced by the persistence of L-LSPR centered in the NIR region. The slight red shift and broadening observed upon silica interaction are consistent with an increased local dielectric environment and partial electromagnetic damping, phenomena widely reported for silica-coated plasmonic nanoshells [18]. This dielectric modulation seems to stabilize the optical response, mitigating excessive inter-particle coupling that can lead to spectral variability in colloidal AuNR assemblies, and that is relevant for intracellular SERS, where signal stability and reproducibility are critical for interpretation in heterogeneous environments [19,20].

Regarding the theranostic performance of AuNR@Si nanoshells, our results demonstrate that this platform enables dual functionality: (i) NIR-triggered photothermal therapy (PPTT) and (ii) SERS-based assessment of treatment response through molecular profiling of early intracellular biochemical alterations. These in vitro studies were conducted in the T47D breast cancer cell line, a widely used model of estrogen receptor–positive (luminal) breast cancer, which represents a clinically prevalent subtype often characterized by heterogeneous therapeutic response and frequent dependence on combination treatment strategies [30,31]. In this context, the 33% reduction in cell viability following AuNR@Si-mediated NIR irradiation, together with the observed loss of cellular integrity, supports that these nanoshells mediate a significant photothermal cytotoxic effect in a hormonally responsive breast cancer model. The accompanying SERS readouts provide molecular-level evidence of treatment-induced damage, consistent with established reports showing that Raman/SERS signatures can resolve intracellular hallmarks of cell death, including protein denaturation, nucleic-acid fragmentation, and mitochondrial perturbation [20].

Beyond therapeutic efficacy, these results have implications for nanoscale detection of malignancy. Raman and SERS techniques have been shown to distinguish malignant from non-malignant cells based on biochemical fingerprints [32], even when morphological differences are subtle or absent [33]. In this context, the preserved NIR-SERS activity of AuNR@Si and its sensitivity to intracellular biochemical changes establish the prerequisites for probing residual or persistent malignancy at micro to nanoscale resolution. Because SERS responds directly to cellular biochemical state, this approach could potentially discriminate whether malignant signatures remain following treatment or are eliminated, complementing conventional bulk viability assays that lack spatial and molecular specificity. However, the efficacy of this approach remains closely tied to particle size, which serves as a critical determinant of cell-nanoparticle interactions. The AuNR@Si nanoshells evaluated in this work exhibit an average diameter of ~193 nm, a size regime that may limit classical clathrin-mediated endocytosis and favor alternative interaction mechanisms such as membrane association, macropinocytosis or sedimentation-assisted contact under static in vitro conditions. Previous studies have shown that nanoparticles above ~150 nm often display reduced internalization efficiency but can still induce strong biological effects through prolonged membrane proximity and localized heating effects [6]. Consequently, the observed photothermal response and SERS-derived molecular signatures likely reflect a combination of surface-associated and partially internalized nanoparticle fractions rather than homogeneous intracellular uptake. This consideration is particularly relevant for intracellular Raman/SERS analyses, which are sensitive to local biochemical environments near membranes and organelles [34]. Future studies will quantitatively assess cellular association and uptake pathways using complementary techniques such as confocal microscopy, flow cytometry, and elemental analysis.

These results support the evidence-based perspective that AuNR@Si nanoshells potentially function not only as effective photothermal agents but also as spectroscopic probes capable of interrogating malignancy-associated biochemical signatures, reinforcing their relevance for integrated cancer theranostics. It is important to note that the present study demonstrates the proof-of-concept theranostic capability of AuNR@Si nanoshells in a luminal breast cancer model (T47D), further validation across additional molecular subtypes is warranted. In particular, triple-negative breast cancer models such as MDA-MB-231 may provide valuable insight into subtype-dependent photothermal and SERS responses. Future studies will extend this evaluation to additional breast cancer subtypes, including triple-negative (e.g., MDA-MB-231) and HER2-positive models, to assess subtype-dependent photothermal and SERS responses.

## 5. Conclusions

We report a mesoporous silica-coated gold nanorod (AuNR@Si) platform designed for dual-plasmonic excitation, enabling integrated photothermal therapy (PTT) and SERS-based molecular monitoring. Through a precise layer-by-layer assembly, the structural anisotropy and dual plasmonic modes of the AuNRs were preserved, allowing for the independent activation of the longitudinal (L-LSPR, 785 nm) mode for therapeutic heating and the transverse (T-LSPR, visible range) mode for diagnostic sensing.

Photothermal characterization revealed that while AuNR@Si exhibits a moderated photothermal conversion efficiency (48%) compared to AuNRs alone (90%), this reduction facilitates superior thermal regulation. Specifically, AuNR@Si sustained the therapeutically optimal hyperthermia range (41–45 °C) for 15 min. These optimized irradiation parameters were successfully translated from solution to in vitro assays, where controlled hyperthermia induced significant molecular alterations (detected via SERS) and a reduction in cell viability to 33%. Furthermore, the silica shell provided plasmonic confinement, enhancing signal stability and reproducibility within the heterogeneous cellular environment. These findings demonstrate that the AuNR@Si platform enables controlled thermal dosing with molecular monitoring of cellular damage, establishing proof-of-concept theranostic functionality in a luminal breast cancer model. Future studies will systematically evaluate the platform across multiple breast cancer phenotypes to assess its broader translational potential.

## Figures and Tables

**Table 1:** Comparative photothermal performance of AuNRs and AuNR@Siunder 785 nm laser irradiation.

Sample	T_max_ (°C)	ΔT_max_ (°C)	PT Efficiency (%)	Time 41–45 °C (min)	PPTT Interpretation
AuNRs	64	39	90	1.24	Rapid heating beyond the therapeutic window, with limited controllability.
AuNR@Si	45	20	48	14.79	Sustained, well-confined heating within the therapeutic range.

**Table 2:** Raman peak associated with PPTT cellular damage.

Peak (cm^−1^)	Assignment	Interpretation
499 ± 1	S–S (disulfide bonds)	Protein degradation
750 ± 1	Cytochrome c (heme breathing)	Cytochrome c release
824 ± 0.5	DNA backbone (O–P–O stretch)	DNA fragmentation
1001 ± 1	Phenylalanine	Protein degradation
1170 ± 0.5	Tyr/nucleic-acid vibrations	DNA fragmentation
1586 ± 0.5	Adenine/Purine bases	DNA fragmentation

## Data Availability

All data generated or analyzed during this study are included in this published article. For data acquiring please send a request to K.S.-G.’s institutional e-mail: karla.santacruz@unison.mx.

## Abbreviations

The following abbreviations are used in this manuscript:

AuNRs	Gold nanorods
AuNR@Si	Hollow silica nanoshells containing gold nanorods
Si	Mesoporous silica
DLS	Dynamic light scattering
EF	Enhancement factor
L-LSP	Longitudinal localized surface plasmon
L-SPR	Longuitudinal localized surface plasmon resonance
T-LSP	Transverse localized surface plasmon
NIR	Near-infrared
PCA	Principal component analysis
PEG	Polyethylene glycol
PPTT	Plasmonic photothermal therapy
SEM	Scanning electron microscopy
SERS	Surface-enhanced Raman scattering
UV–Vis	Ultraviolet–visible spectroscopy
ζ-potential	Zeta potential
ΔT	Temperature increase
ΔTmax	Maximum temperature increase
η	Photothermal conversion efficiency
